# Pilot implementation of community health advocacy teams to improve the effectiveness of long-lasting insecticide net distribution through both campaigns and continuous channels in Ghana: a qualitative study of opportunities and barriers to implementation

**DOI:** 10.3389/fpubh.2023.1133151

**Published:** 2023-07-31

**Authors:** Phyllis Dako-Gyeke, Ruby Hornuvo, Franklin N. Glozah, Emmanuel Asampong, Philip Teg-Nefaah Tabong, Adanna Nwameme, Gloria. M. Chandi, Nana Yaw Peprah, David Gittelman, Philip B. Adongo

**Affiliations:** ^1^Department of Social and Behavioural Sciences, School of Public Health, University of Ghana, Accra, Ghana; ^2^Ghana Health Service, Ga North Municipal Health Directorate, Accra, Ghana; ^3^National Malaria Elimination Programme, Accra, Ghana; ^4^Health Campaign Effectiveness Coalition, Task Force for Global Health, Decatur, GA, United States

**Keywords:** community health advocacy team, implementation research, intervention, long-lasting insecticide net, malaria, Ghana

## Abstract

**Introduction:**

In Ghana, the National Malaria Elimination Programme (NMEP) distributes long-lasting insecticide net (LLIN) to households for free through the periodic point mass distribution (PMD) campaign and continuous distribution to populations most vulnerable to malaria. It is known that the existence of effective and functional community-based groups could influence positive behaviours regarding health interventions promoted through health campaigns. However, there is no evidence of functional community-based groups that aim to improve the effectiveness of LLIN distribution campaigns by transitioning into primary healthcare delivery. This study aimed to explore the opportunities and barriers to the pilot implementation of co-created community health advocacy teams (CHATs) to improve the effectiveness of LLIN distribution through both campaigns and continuous channels in Ghana.

**Methods:**

A qualitative research approach was used among 43 CHAT members across six communities in the Eastern and Volta regions of Ghana. The CHAT constitutes significant community actors whose roles are centred on key elements of community/social mobilisation and capacity building, all nested in social and behaviour change communication (SBCC) strategies. The CHATs were pilot implemented in all study communities for 4 months after which we identified opportunities and barriers during implementation. CHAT members participated in six focus group discussions which were audio recorded, transcribed verbatim, and analysed thematically using the NVivo 13.

**Results:**

CHATs were instrumental in sensitising community members through SBCC strategies. Moreover, there were changes in the behaviour of community members who were receptive towards and participated in CHAT activities. Community members were accurately informed about malaria (e.g., causes and preventive measures). However, the CHAT experienced barriers during implementation, including a lack of financial support to aid in transportation, organisation of meetings, and outreach activities. Additionally, the level of participation by CHAT members in activities and the medium of communication among members were key areas of concern.

**Conclusion:**

The CHATs would be instrumental in promoting LLINs' use during and after PMD campaigns through community outreaches. It is therefore necessary to provide resources to support their operations and a good network to address communication barriers. Finally, continuous capacity strengthening of CHAT members by the NMCP is important.

## 1. Introduction

Malaria is a public health concern with nearly half of the world's population at risk of infection, and the major cause of morbidity and mortality in many resources constrained settings especially for children under 5 years (1). The World Health Organisation (WHO) has recommended long-lasting insecticide-treated nets (LLINs) as a core intervention in all malaria-endemic settings. The LLIN is estimated to reduce malaria cases by 50% (2). To reduce the global burden of malaria by 90% by 2030, WHO advises universal coverage with effective vector control utilising LLINs and indoor residual spraying (IRS) for all persons in malaria-endemic areas (1).

Malaria is a parasitic and infectious disease caused by *Plasmodium*. The parasite is transmitted through the bite of an infective female *Anopheles* mosquito during a blood meal from one person carrying the parasite to the other. The main vectors of malaria in the country are *Anopheles gambiae* complex and *Anopheles funestus* group. In Ghana, malaria is mainly caused by the *Plasmodium falciparum* parasite, which is responsible for >85% of malaria cases. The other malaria parasites are *Plasmodium malariae* and *Plasmodium ovale*. Because the malaria parasite is found in the red blood cells of an infected person, malaria can also be transmitted through blood transfusion, organ transplant, or the shared use of needles or syringes contaminated with blood. Malaria may also be transmitted from a mother to her unborn infant before or during delivery (“congenital” malaria). Malaria is hyper-endemic in Ghana with transmission occurring year-round, and the peak transmission occurring between June and October (rainy season).

The Ministry of Health (MoH) in Ghana oversees healthcare organisations in Ghana and this includes public, private, or traditional ownership in the country. The Ghana Health Service (under the MoH) is a public service body that provides and supervises public healthcare in the country. It has eight directorates that include the National Malaria Elimination Programme, regional and district health administration, and subdistrict health administration, which includes Health Centres and Community-based Health Planning and Services (CHPS).

The National Malaria Elimination Programme (NMEP) in Ghana is responsible for mass LLIN distribution campaigns by engaging and involving stakeholders at all levels (national, regional, district, sub-district, and community) (3). In accordance with Ghana's Malaria Strategic Plan (2021–2025), the mass LLIN distribution campaign seeks to protect at least 80% of the population at risk with effective malaria prevention interventions through household registration (90%) and distribution (90%) in target regions (4). Over the years, the NMCP together with its partners continues to scale up the LLIN ownership through point mass distribution (PMD). As part of efforts to achieve universal coverage of LLINs, continuous distribution of LLINs to the population most vulnerable to malaria (i.e., pregnant women, mothers of children under 5 years, and primary school children) is done through antenatal care clinics (ANC), child welfare clinics (CWC), and schools. From 2010 to 2012, there was a nationwide LLIN door-to-door mass distribution and the hang-up campaign, which was followed by another mass distribution campaign in 2018. Despite progress in overall LLIN ownership, the challenge remains to reach the NMCP strategic plan target of 80% usage among pregnant women and children under 5 years. Moreover, the 2019 Ghana Malaria Indicator Survey shows that 67% of Ghanaian households have access (percentage of the population that could sleep under an LLIN if each LLIN in the household were used by up to two people) to LLINs, but only 43% of Ghanaian household population slept under a net the night before the survey (3). This indicates that a relatively large number of people have not used the LLIN despite the distribution campaign. Although these campaigns have exposed a large proportion of Ghanaians to LLINs, they may not have led to desired health-related behaviours (i.e., sleeping in LLINs every day).

Various studies have documented barriers to LLIN use, which include inadequate distribution of nets per household, limited social and behaviour change communication (SBCC) activities to support distribution, lack of malaria education on the proper use of LLINs, and complaints of nets being distributed to communities with little or no information on their relevance for malaria prevention (5–7). Furthermore, LLINs are not used following complaints of burning sensation or itching from sleeping under the net and inconvenience due to heat. At the community level, LLINs are sometimes inappropriately used for gardening/fencing, fishing, crop farming, and processing of farm produce (6, 7). The inability to hang LLINs due to housing type and sleeping places has been observed in other communities. Barriers that health workers experience include a lack of community mobilisation training, inadequate personnel, lack of follow-up, involvement, and supervision (8, 9).

To achieve national LLIN access and use targets, innovative social interventions that facilitate behaviour change may be needed both during and in follow-up to campaigns (10). Social innovation is described as a collaborative approach that generates ideas to improve community or hospital delivery systems (11). Social purpose emphasises engaging concerned communities within which innovative approaches fulfilling both social and health concerns will be distributed (11). Such community-based programmes allow the government, health agencies, social actors, and individuals to work closely with populations impacted by diseases, especially infectious conditions. The Community-based Health Planning and Services (CHPS) in Ghana is a national-level programme that aims to provide accessible, equitable, efficient, and high-quality healthcare (12). The CHPS programme is considered a pragmatic strategy for achieving universal health coverage of a basic package of essential primary health services. The CHPS concept involves the provision of door-to-door primary healthcare services to community members by trained nurses known as community health officers (CHOs) and has proven to be successful in providing maternal, reproductive, and child health services in communities where they are much needed (12–14). CHOs provide antenatal care, family planning, health education, outreach clinics for delivery of child welfare services, and school health services. Some community health workers (e.g., health volunteers and community health nurses) are involved in household registration and distribution of LLINs during the PMD campaigns, after which they are remunerated for their work. These community health workers may not necessarily be the ones mandated to engage in LLIN promotion and use both during and after campaigns.

In order to use a person-centred approach to promote LLIN use which leverages CHPS and ensures community involvement, ownership, and sustainability of the LLIN mass distribution campaigns, a community health advocacy team (CHAT) was co-created in six Ghanaian communities (15). The terms of reference of the CHAT are generally based on NMCP's key elements of the campaign at the sub-district level (e.g., household registration, training, SBCC, and logistics). Specifically, the CHAT members should be equipped with skills in community mapping; promoting correct LLIN use, maintenance, and repurposing; leadership and supervision, record-keeping, and interpersonal and persuasive communication. This study explores the opportunities and barriers to the pilot implementation of co-created CHAT in Ghana. The goal is to transition the community-level LLIN ownership and use promotional functions provided during the PMD Campaigns with ongoing LLIN promotion post-campaigns for continuous distribution under the Community Health Planning and Services (CHPS) programme.

## 2. Materials and methods

### 2.1. Study design

This study used a qualitative research approach to explore the opportunities and barriers experienced by CHAT members in a pilot implementation of the intervention. A total of six districts (one community per district) across two regions in southern Ghana participated in this study. These were communities in districts where the 2021 PMD campaigns of LLINs were ongoing. These communities were selected to avoid possible biases concerning community engagement (i.e., communities that are yet to be involved in registration and distribution activities for the 2021 PMD campaign) by ensuring that components align with the timelines of the National Malaria Elimination Programme (NMEP) and the funder. The study was also conducted in districts with the highest malaria prevalence as reported in the District Health Information System: Ho West (Tsito-−90%), Ho (Takla Hokpeta-−75%), and Agortime Ziope (Kpetoe-−100%) in the Volta Region; and Birim South (Apoli-−94%), Achiase (Achiase-−94%), and Abuakwa North (Kukurantumi-−93%) in the Eastern Region (Data source: DHIMS 2). At the time of the study, continuous/routine LLIN distributions in schools and antenatal care clinics were ongoing in these communities.

### 2.2. Population and sample

**Figure 1:**
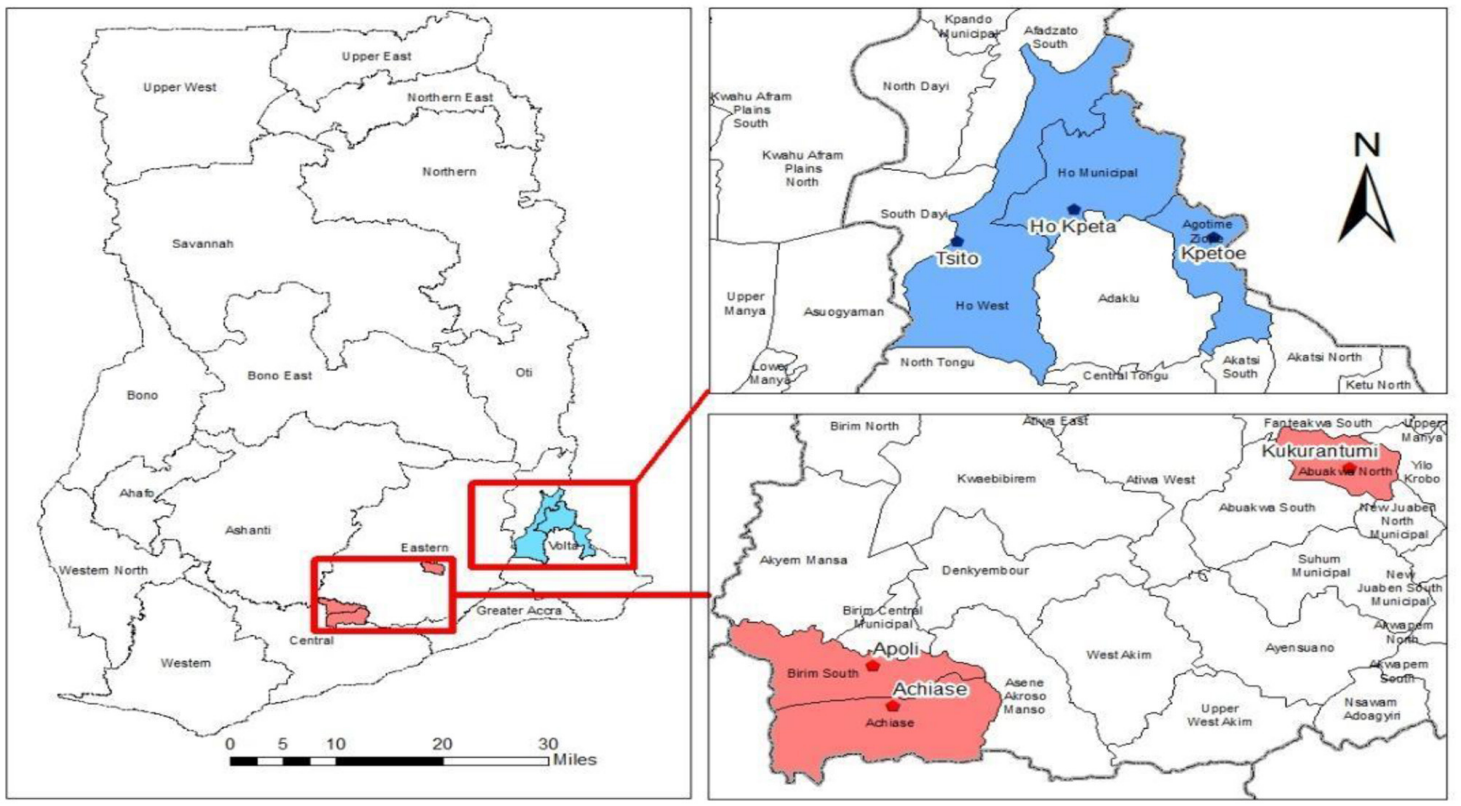
Location of the six districts in the Eastern and Volta Regions of Ghana.

The study population comprised adult men and women from communities within the selected districts across two regions in southern Ghana (Eastern and Volta Region). The sample consisted of 43 members (18 women and 25 men) of the CHAT from six communities in the two regions (Figure 1).

#### 2.2.1. The community health advocacy team

The community health advocacy team (CHAT) was co-created through the participatory learning in action technique using participatory workshops (PWs) which is a practical approach. This approach involves adaptive research strategies that enabled diverse groups and individuals to learn, work, and act together in a co-operative manner, to focus on issues of joint concern, identify challenges, and generate positive responses in a collaborative and democratic manner. This was done by using the findings from the initial phases of the project (i.e., desk review, focus group discussions (FGDs), key informant interviews (KIIs), and baseline surveys) (15). The participatory workshops involved various stakeholders (i.e., project investigators, NGO representatives, school health education programme coordinators, ANC nurses, disease control officers, district health management teams (DHMTs), CHOs, community leaders, and opinion leaders). Findings from the PWs suggested the establishment of a CHAT can be instrumental in facilitating and improving the effectiveness of LLIN distribution campaigns within communities in Ghana.

A CHAT consists of nine members who are influential in their communities: health officers, religious leaders, school health education programme coordinators, assemblymen/women, community information officers, representatives from any of the security services, community-based organisations, and traditional authorities.

The CHAT members were trained by officials from the NMCP and project investigators as part of their capacity-strengthening efforts. They were trained in key elements of the NMCP's campaign (i.e., training, registration, SBCC, logistics, distribution, and supervision) and skill-enhancing strategies in leadership, communication, and community mapping, as well as record-keeping competencies. This training provided CHAT members with the capacity to carry out malaria education and prevention activities as well as the promotion of net use within communities and primary healthcare levels during and after LLIN campaigns. Specifically, CHAT members are expected to support PMD for LLIN campaigns at the community level, as well as provide support on the continuous distribution of LLINs through the school-based, antenatal, and child welfare clinics at the community level, development of context-based social and behavioural change communication (SBCC) strategies on malaria prevention and regular use of LLINs, sensitise the community on the proper use of LLINs and its maintenance, support with the management of LLINs logistics and accountability, and support other community-based health campaigns. All stakeholders agreed that the CHAT would meet quarterly to discuss implementation progress and re-strategizing as needed.

After successful training of the CHAT, a total of six community health advocacy teams, one in each of the six districts, were inaugurated and out-doored (an introduction of CHAT to community members for the first time) at an organised community durbar (an outdoor community gathering, where members of the community are present to discuss issues of community importance). These community durbars included traditional leaders, community members, religious leaders, opinion leaders, representatives from the Ghana Health Service (Regional Deputy Director of Public Health (DDPH), District Director(s), regional and district malaria focal persons, CHAT members, project investigators, and representatives from the NMCP) in the six study sites.

#### 2.2.2. Long-lasting insecticidal net

**Figure 2:**
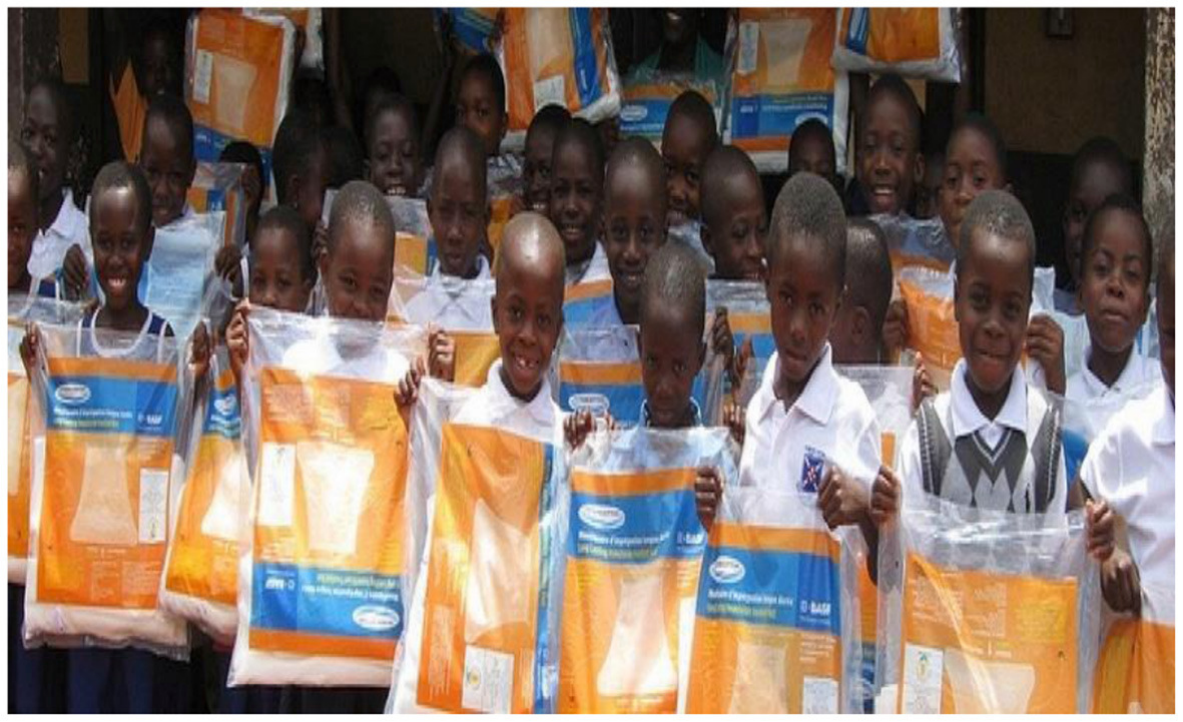
School children receiving treated mosquito net in Ghana. Source: Malaria consortium.

**Figure 3:**
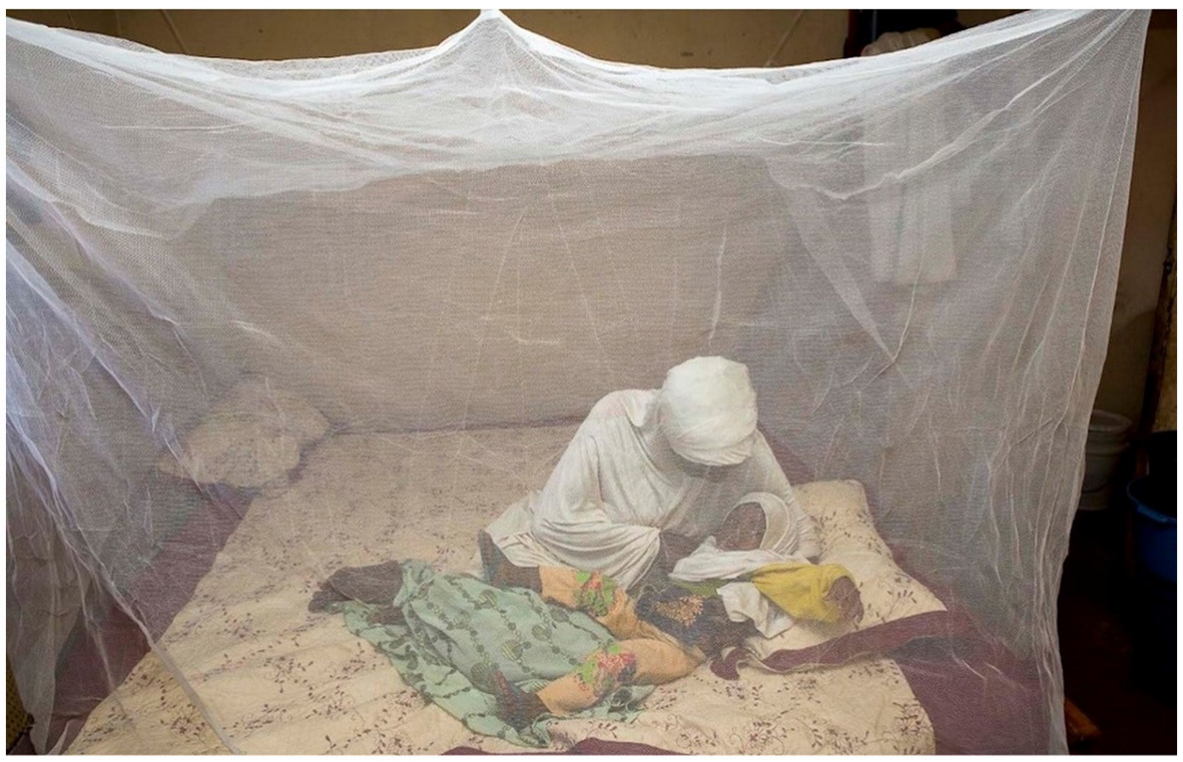
Mother and child sleeping under treated mosquito net. Source: WHO Africa.

**Figure 4:**
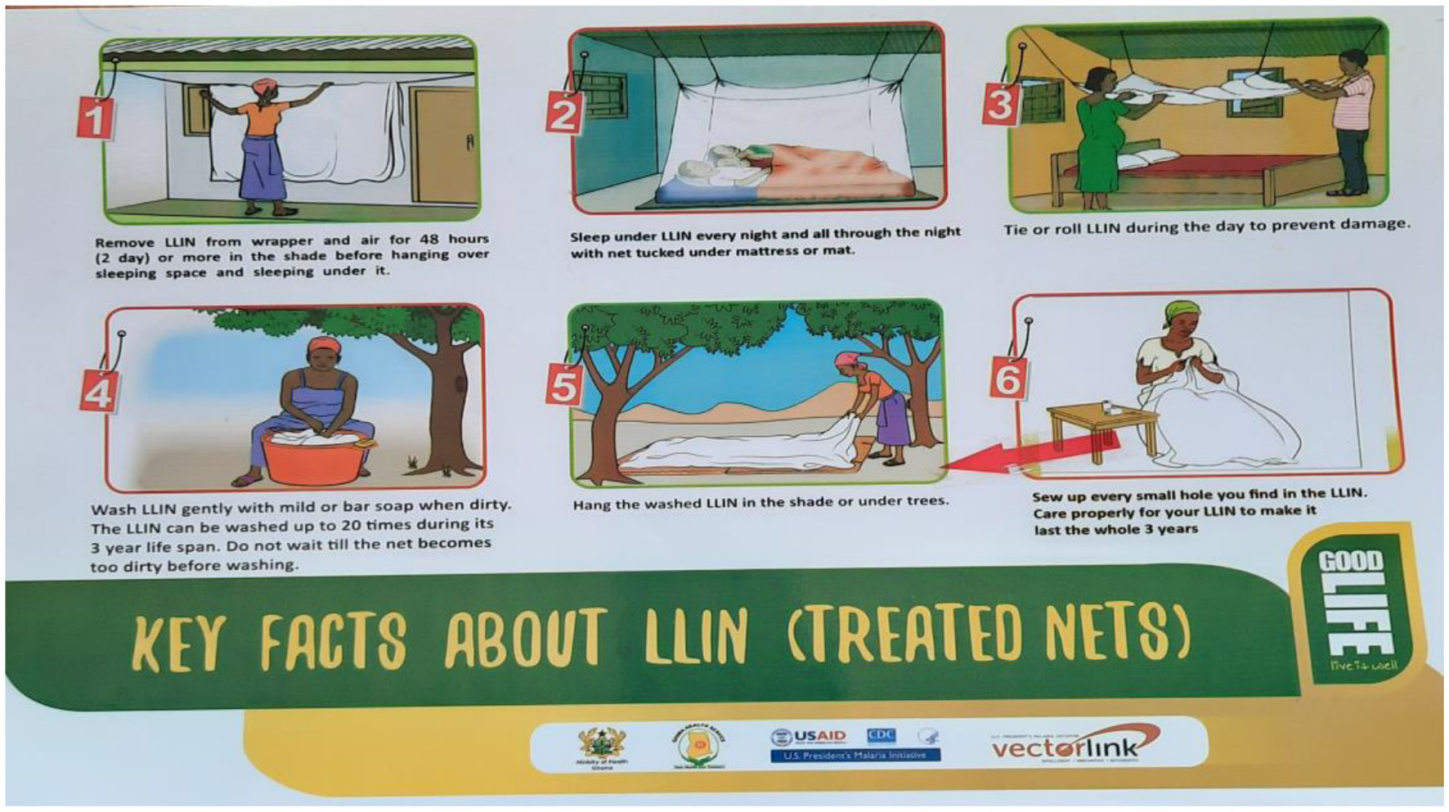
Demonstration of LLIN use, care, and maintenance. Source: Ghana Health Service.

The NMEP is responsible for reducing malaria morbidity and mortality in Ghana and has, over the years, carried out several malaria prevention interventions such as PMD of LLINs (Figure 2). The distribution and the use of LLINs are core interventions for preventing malaria infection in malaria-endemic countries, including Ghana. LLINs provide protection against mosquito bites, repel, and kill mosquitoes, thereby reducing the transmission of malaria parasites and decreasing malaria risk at the individual and community levels when high coverage is achieved (Figures 3, 4). Mosquito nets can be obtained mainly during PMD campaigns; however, as part of targeted continuous distribution programmes, LLINs are distributed through antenatal care (ANC), child welfare clinics (CWC), and primary schools. LLIN can be used for up to 3 years or after 20 washes.

### 2.3. Data collection

A total of six focus group discussions (FGDs) were organised to explore the opportunities and barriers of the CHAT intervention after four months of pilot implementation in all six study communities. Each FGD included members of the CHAT, with a total of 43 participants in all six FGDs. Some CHAT members were playing key roles at a traditional function during the time of data collection and hence could not participate. All participants were contacted with the assistance of the conveners of the team and an arrangement was made for the FGDs to be conducted. The FGDs were conducted by trained qualitative research assistants using a designed implementation stage FGD guide within a relaxed and convenient atmosphere while observing all COVID-19 protocols. The interviews were conducted by experienced research assistants who have been trained in public health and with several years of conducting qualitative research and interviews. Saturation was achieved during interviewing as similar themes emerged repeatedly in the course of the interviews. Informed consent was sought from all participants and FGDs were audio-recorded. Each FGD lasted approximately one hour.

### 2.4. Data analysis

All audio-recorded FGDs were transcribed verbatim and augmented with researchers' field notes made through observations and during FGDs. A codebook was developed based on the research objectives. The codebook development involved qualitative experts from the project team who reviewed the various components of the codes to ensure they aligned with the datasets. The data resulting from transcriptions were evaluated, coded, and analysed using the thematic analysis method, employing both deductive and inductive processes as described by Braun and Clarke (16). The data were analysed thematically and managed using the NVivo software version 13. The initially developed codebook was revised throughout the coding process to include emerging codes. The consolidated criteria for reporting qualitative studies (COREQ): 32-item checklist was used to guide the process.

### 2.5. Ethical consideration

Ethical clearance was obtained from the Ghana Health Service Ethics Review Committee (GHS-ERC: 002/06/21) before the commencement of all data collection. All research assistants received specific training before data collection as per the study's training protocol.

Before beginning, all study participants provided written informed consent after reviewing the study aim, procedures and benefits, and their rights as participants. The information and consent documents for participants were written in simple English. However, for better comprehension, research assistants were present during the informed consent process to explain any questions that the participants do not understand. Those consenting to participate either signed or placed a thumbprint on an informed consent form. All participants were assured that the information they provided would be handled confidentially and research findings would be reported with complete anonymity.

## 3. Results

The findings of the study are presented under the following headings: sociodemographic characteristics of participants, CHAT implementation opportunities [increase LLIN use and malaria prevention (malaria control interventions) through CHAT educational and SBCC activities, community participation in CHAT activities], and CHAT implementation barriers.

### 3.1. Sociodemographic characteristics of participants

A total of 43 participants comprising 18 women and 25 men, aged between 23 and 73 years, were involved in the focus group discussions. Participants were the CHAT members from the selected districts in the Eastern Region (Kukurantumi, Achiase, and Apoli) and Volta Region (HoKpeta, Tsito, and Kpetoe) of Ghana. Table 1 presents the socio-economic characteristics of the participants (*NB: R* = *Respondent/Participant*).

**Table 1:** Sociodemographic characteristics of participants.

**Characteristic of participants**	**Number of participants**
**Region**	
Eastern Region	27
Volta Region	16
**Total**	**43**
**Sex**	
Female	18
Male	25
**Total**	**43**
**Age**	
20–29 years	4
30–39 years	17
40–49 years	8
50+ years	14
**Total**	**43**
**Educational Level**	
Primary	2
JHS/Secondary/Middle School	10
Tertiary	31
**Total**	**43**
**Marital Status**	
Single	15
Married	28
**Divorced/Widowed/Separated**	
**Total**	**43**

### 3.2. CHAT implementation opportunities

CHAT implementation opportunities were assessed both during the campaign and afterwards in continuous distribution mode. These opportunities include sensitisation on the use of LLIN and malaria prevention, as well as community participation in CHAT activities were explored.

#### 3.2.1. Sensitisation on the use of LLIN and malaria prevention

Findings from the study revealed that the CHAT was able to promote LLINs use and sensitise community members on malaria prevention strategies during and after the 2021 mass LLIN distribution campaign. The CHAT members provided education on malaria prevention to various target groups such as mothers of children under five years, primary school children, and household members. For instance, the CHAT provided mothers of children under 5 years, at the CHPS level, with education on the proper use of LLINs and malaria prevention:

“*We organised and have Child Welfare Clinic (CWC) every time, there we demonstrated to the mothers how to use the net. Because we got to know[sic] most of the kids are coming to the hospital with malaria, so[sic] we saw that the malaria cases are still going high with the kids now. It's[sic] no more with the adults much like that. So, we came to demonstrate to them how the mosquito net is being used.” (R5, Tsito, Volta Region)*

“*CWC. We all do that because the volunteer also helps in the organisation of the people. We entreat the pregnant woman to sleep under the mosquito net. We talk to them about the causes of malaria and that sleeping under the treated net will help prevent malaria.” (R3, HoKpeta, Volta Region)*

In addition, the CHAT also engaged school children and community members during religious gatherings, and this appears to be a good platform because school children are likely to spread information from the school to members of their households. Moreover, as Ghanaian communities are largely religious, it is a good platform to communicate health messages. For instance, some participants highlighted that

“*For the school, I talk to the kids about mosquito nets and I ask them if their parents are using them and their response is always yes. I think they were given just last year so according to them, they are using it. So far, so good.” (R3, Tsito, Volta Region)*

*So far, we have been to schools to give education about the prevention of malaria.” (R2, Kukurantumi, Eastern Region)*

“*After our inauguration, it was almost in the festive season, that is Christmas, and the New Year was approaching, so we decided to take that opportunity to meet the large crowd” [at various churches]. (R5, Apoli, Eastern Region)*

“*We educate them. When we go to church after we close, we also remind them on the use of the mosquito net and their children under 5 years should also sleep under and also after worship on Wednesdays.” (R4, HoKpeta, Volta Region)*

Home visitation is another effective way to communicate health messages to achieve desired behaviour changes. The findings further revealed that the CHAT engaged community members on a one-on-one basis during home visits to educate community members on the need to use the mosquito nets, so as to achieve the intended purpose:

“*As a community volunteer, when I am walking within the community and see they are using the net for the wrong purpose, I talk to them to use it the right way, sleep under it in order to prevent malaria.” (R2, HoKpeta, Volta Region)*

The study also showed that CHAT members employed various SBCC channels in delivering malaria messages to community members. Mediums, such as the community information centres (CIC) and banners, were mentioned. For instance, some participants mentioned that

“*We use the CIC, the local information centres. We have radio stations too-[sic] we engage them and durbars as well. Any gathering we get, we chip in and say something as well****.” (****R4, Kukurantumi, Eastern Region)*

“*When we go, we educate with the SBCC materials as we said; our posters are showed[sic] to them as they see and they remember.” (R6, Apoli, Eastern Region)*

“*With CIC Representative, you know he is into the broadcasting and as a member, he took that one up and you know we don't pay again to him and it's the service to the community. So, every Wednesday, they do come for the early morning program. That is at 5:30 so by then people will wake up and listen to worship and after that, they come in mostly about the mosquito net issue and the prevention of malaria... every Wednesday. Every Wednesday they do it unless maybe if[sic] when there is no resource person on Wednesday.” (R1, Tsito, Volta Region)*

Use of social media platforms could have been a faster way of promoting malaria prevention messages. However, some participants indicated that poor network access prevented use of social media in some communities.

“*You see, there is no network here, so those who live here will have difficulty in downloading and watching the video but those of us in Ho can easily view it.” (R3, Hokpeta, Volta Region)*

#### 3.2.2. Community participation in CHAT activities

The findings also revealed that there have been perceived changes within the various communities concerning malaria prevention and LLIN use that can be attributed to the malaria education activities that the CHAT has been engaged in. For instance, some CHAT members mentioned that there has been a perceived reduction in malaria cases within the communities:

“*In fact, in my place, it has been a long time now that I have heard of malaria. Even in my room, my children are using it, so that one is out. So, as we said, before we can see the actual result, it will be the statistics. But for me, we believe that even when you ask people. It's just the young ones who are reluctant in using it. But older adults and women are using it.” (****R1*, ***Tsito, Volta Region)*

In addition, there has been a positive reception from community members towards CHAT education activities on malaria prevention:

“*In schools, knowing the presence of this group, they are paying more attention. The misuse of the nets is not good for both teachers and pupils. Also, we told them that there is a committee, like the security personnel, to cheque on the misuse of the net…As we went to some school[sic, the children came asking individually; if the net is torn and if it is used otherwise, they also will be caught?[sic] The madam continued that they will be interrogated about when they were given and the number of times it has been used that will show whether it is due to be thrown away or not. So, they are now serious about the dump of the nets****.”*** *(R3, Achiase, Eastern Region)*

“*When we go for gathering and we announce that we are coming and you see lots of the community members seated and during funerals and the outreach we had too, they were very attentive and will do what we say****.”*** *(R2, Kpetoe, Volta Region)*

Active participation in CHAT activities may have helped change in behaviour among community members, as they observed improved understanding of malaria prevention and LLIN use.

“*The education we have been doing has been valued because when we visit the back of some houses, there is now neatness. Those we educated are now aware and now everyone is tidying their surroundings up because dirt brings malaria so what we have been educating has been valued and the elders have seen it will help and their expenses will be cut short because a single mosquito bite can cause serious loss of income before you get healed[sic]. So, if the mosquito net can solve that problem, anytime, everyone should try and use the mosquito net. Malaria is reducing and we hope that it won't be long for it to be curbed.” (R2, Achiase Eastern Region)*

“*Yes, we have got many moments as we visit churches and gatherings where we have our health talk, misunderstandings about the[sic] malaria are being discussed as to the cause of malaria as some are misinformed that malaria is from the sun.” (R5, Apoli, Eastern Region)*

“*Some also said that malaria is brought by unripe mangoes so some people with these misconceptions, we speak to them to inform them that it is mosquito bites that cause it.” (R8, Apoli, Eastern Region)*

“*What we can say is, OPD cases attendance has increased. So, with that, we can justify by saying that, maybe because of the sensitisation going on home management and those things are reducing and they are now visiting the facilities to seek treatment.” (R6, Kpetoe, Volta Region)*

### 3.3. CHAT implementation barriers

Some barriers that the CHAT faced within the four months after their inauguration were discussed during the discussions. For instance, some members said that the medium of communication and active participation by members at CHAT meetings to plan and strategise activities have been a major challenge:

“*We were using the WhatsApp page, and some people may not even have the data and even at Takla, the network is not all that good so we have to be calling everyone. So, it is very difficult.” (R2, HoKpeta, Volta Region)*

“*Also, like the heads, every position has its duty to undertake like ‘Madam A', is part of us (CHAT). Like for the Friday[sic] she didn't go to the farm. Saturdays, there are funerals, and on Sundays people go to church. She was supposed to go to the farm yesterday but there was another meeting she was supposed to attend so she wasn't able to [sic] join us (CHAT meeting). And for me too, sometimes I have other tasks to take so I have to stop (the CHAT meeting) and attend the other meeting. So, these are the challenges we are facing.” (R2, Apoli, Eastern Region)*

“*Yes, when you call for a meeting, not even a meeting, when you post something on the page, for people to respond to it, it becomes a problem. It is as a result [of lack] of motivation; that the motivation level has fallen. So, people feel like, I need to go somewhere to make (some money) than to have attention here so that is it...So I think being frank with you[sic], it will help all of us.” (R3, Kpetoe, Volta Region)*

Moreover, the need for some form of support in terms of transportation to undertake outreach activities, especially in hard-to-reach communities was mentioned by members.

“*Yes, going for programmes, there are some costs that come with it such as the transportation and maybe feeding. All these are on the individuals and sometimes, the Madam will have to come in by buying water for the people. So, these are barriers and we need to fix them.” (R3, Kpetoe, Volta Region)*

“*The main challenge now is our means of transport unless like we were going round, I was the one who bought petrol to all the six churches to the other places. Also, in other places, we let them meet in a congregation, then I pick them up. Currently, the earth has also been challenging where you take someone out for hours and you don't give them [sic]chance at their work, it feels bad though this is just the beginning of the team but after that, everyone can return to their workplaces after success. So, our challenge is as we are planning to visit another town like Aprade and other places, we need means for[sic] transport to support as[sic] like getting us petrol so we don't charge anything- we can just go and come back.” (R4, Achiase, Eastern Region)*

“*…and like places like Yaw Agbo, going there, we have to take the motorbike and most of us don't have the motorbike, so unless we hire and that also sometimes it's hard to.” (R3, Apoli, Eastern Region)*

“*Like today as we were going to the school, we paid the fare of the transport. The last time we went, we were supposed to go in a group but already some were members so we had to pay for the fare.” (R6, Kukurantumi, Eastern Region)*

Similarly, participants expressed the need for the provision of refreshment during outreach activities as most of the outreach activities take long hours and they need some water or food during these outreaches.

“*There is no addition really, but if you can be supporting us small, small- at least water will do.” (R4, Hokepta, Volta Region)*

“*… and probably if we get something small like lunch or minerals, though they are adults but they know what they are doing so they are not demanding anything. So, they need something small to support them like small snacks like biscuits, that is our challenge.” (R4, Achiase, Eastern Region)*

“*Also, we would be needing water and snacks so that inclusive will help because some from morning do not eat when going for education in the other towns. So at least, water should be provided.” (R7, Achiase, Eastern Region)*

The need for some financial support in carrying out outreach activities was also mentioned.

“*Our main problem is the financial aspect where resources are needed like inviting professional resource personnel to come educate us, we need money to invite them too. Sometimes we volunteer and take on such tasks…we also plan to involve other members like the midwives and some doctors so they can join in the education. But afterwards, we have to pay them, which might turn into another challenge.” (R2, Kukurantumi, Eastern Region)*

In addition, some CHAT members reported the need for further instruction on transmitting messages to communities on malaria prevention and correcting LLIN misuse. Notably, some indicated that

“*I once met a lady like that, upon probing, she said, they are very old mosquito nets that are over 7 years old and are worn out and can't be used, that is why, she used them for gardening/fencing. And I couldn't say anything again because 7 years is a long time. So such people too, what do we do to them[sic]?” (R3, Hokpeta, Volta Region)*

“*I will say we need some motivation (e.g., capacity building) in relation to the net distribution. Some people complain about the net. Let's say they are five (5) in the house and maybe only two (2) were able to get the nets so the rest explain to them that okay maybe there was distribution in the various schools so when the child gets it in the school, they can use it at home. So, we need to know it and explain [sic]to them so that every household can get it.” (R1, Kpetoe, Volta Region)*

## 4. Discussion

This study explored the opportunities (LLINs use and malaria prevention strategies, community participation in CHAT activities) and implementation barriers to the pilot implementation of the co-created CHAT. These teams seek to integrate the LLIN distribution campaigns with the CHPS programme to promote community LLIN ownership and use both during and beyond campaigns in Ghana. This study has yielded important findings that will help provide the CHATs with the necessary support to effectively perform their roles and address barriers. The findings can also inform further scaling-up of the CHATs across Ghana.

The study showed that CHAT members engaged different groups of the population during their malaria prevention sensitisation. The CHAT members employed SBCC strategies to educate them on the continuous use of the treated mosquito nets. As evidence suggests, the use of SBCC can improve malaria prevention and treatment behaviours (10, 17). The employment of various mediums (e.g., community information centres) and platforms (e.g., child welfare clinics, schools, religious gatherings, and door-to-door visits) serves as a means of reaching a larger population with malaria prevention messages which will help contribute to achieving sustainable outputs and impacts concerning malaria control in Ghana (18–21).

Community reception is key to achieving desired health behaviours (20). The study revealed that community members were receptive to the CHAT members whenever they were carrying out malaria sensitisation activities. Moreover, community members became better informed about the causes of malaria, malaria prevention strategies, as well as the use of LLINs for the intended purpose. Community members were also taught how to properly hang the nets regardless of housing style or sleeping places. Possible positive changes in community behaviour are very important as they address some barriers to LLIN use that have been realised in earlier studies such as limited use of SBCC activities, lack of continuous malaria education (5, 22–24), knowledge gap at the community level on malaria prevention, inability to hang LLINs in many household types and sleeping places, and the misuse of nets (6, 7). Access to accurate information about malaria can promote increased use of LLINs and reduce the gap between LLIN access to and use eventually contributing to the reduction in malaria morbidity and mortality.

Although the pilot implementation of the CHAT has achieved many successes, it encountered several barriers. Most of these barriers revolved around financial support in carrying out voluntary activities. Although the CHAT has been set up as a voluntary team without any remuneration, there were situations where they needed financial resources to carry out LLIN and malaria sensitisation activities. Some of these activities include transportation to neighbouring communities or hard-to-reach areas of communities as well as providing refreshments after community outreach activities, as they usually spend long hours during these outreaches. These findings are consistent with other studies conducted in other parts of Africa, where a lack of financial support is reported to impede the delivery and sustainability of health volunteer work (25–27).

## 5. Limitation

Although this exploratory study of the opportunities and barriers in the pilot implementation of the CHAT provides some useful lessons, the four-month duration of the pilot implementation is relatively short to unravel all possible lessons. There is a need for further scale-up beyond the six pilot districts in Ghana to assess its effectiveness and impact with respect to LLINs promotion and use. Moreover, due to the seasonality of malaria transmission in Ghana (i.e., during rainy seasons—April to June and September to November), the season within which the CHAT was implemented could also influence the opportunities and barriers realised; hence, there is the need for long-term implementation of the CHAT to effectively observe the seasonal variations of opportunities and barriers of CHAT in promoting LLIN use and malaria prevention.

## 6. Conclusion

The community health advocacy teams have a great promise to sustain community LLIN promotion activities both during PMD campaigns and afterwards in relation to the primary healthcare system in Ghana. In order for this to happen, there is the need to address barriers to the effective functioning of the CHATs, including the provision of financial support to aid transportation, the provision of refreshments and support for their outreach activities, the provision of financial motivation to increase their level of participation in CHAT activities, and the provision of good network access to address their communication barriers. Finally, CHAT members require continuous capacity building, especially in use of SBCC to promote LLIN access and use, to most effectively support the PMD and to transition LLIN distribution to routine or continuous channels through CHPS.

## Data availability statement

The raw data supporting the conclusions of this article will be made available by the authors, without undue reservation.

## Ethics statement

The studies involving human participants were reviewed and approved by Ghana Health Service Ethics Review Committee. The patients/participants provided their written informed consent to participate in this study.

## Author contributions

PD-G, FG, EA, PT, and AN were in charge of conceptualisation, data curation, formal analysis, methodology, and original draft. RH supported with data analysis, methodology, and drafted the manuscript. PA and DG reviewed and revised the final draft. All authors participated in designing the study with NP and GC providing technical support. All authors contributed to writing the manuscript and approved the final draft.

## References

[1] World Health Organization. Guidelines for Malaria Vector Control. Geneva: World Health Organization (2019). 30844152

[2] Dzata ST Coleman N Quakyi I. Coverage and use of long-lasting insecticide treated nets in Kpone-on-Sea Township, Accra, Ghana: a cross-sectional study. Heal Sci Investig J. (2020) 1:57–63. 10.46829/hsijournal.2020.6.1.1.57-63

[3] Ghana Statistical Service (GSS) and ICF. Ghana Malaria Indicator Survey 2019. Accra; Rockville, MD: GSS and ICF (2020).

[4] WHO U. Achieving the Malaria MDG Target: Reversing the Incidence of Malaria 2000–2015. Geneva: World Heal Organ. (2015)

[5] Worrall E Were V Matope A Gama E Olewe J Mwambi D. Coverage outcomes (effects), costs, cost-effectiveness, and equity of two combinations of long-lasting insecticidal net (LLIN) distribution channels in Kenya: a two-arm study under operational conditions. BMC Public Health. (2020) 20:1–16. 10.1186/s12889-020-09846-4 33287766 PMC7720381

[6] Opoku R Amoah PA Nyamekye KA. Householders' perception about sustaining the useful life of long-lasting insecticide-treated nets in Ghana. Int Health. (2021) 13:57–62. 10.1093/inthealth/ihaa019 32497203 PMC7807238

[7] Bannor R Asare AK Sackey SO Osei-Yeboah R Nortey PA Bawole JN. Sleeping space matters: LLINs usage in Ghana. Pathog Glob Health. (2020) 114:271–8. 10.1080/20477724.2020.1776920 32530747 PMC7480583

[8] Assan A Takian A Aikins M Akbarisari A. Universal health coverage necessitates a system approach: an analysis of Community-based Health Planning and Services (CHPS) initiative in Ghana. Global Health. (2018) 14:1–10. 10.1186/s12992-018-0426-x 30413209 PMC6230285

[9] Malede A Aemero M Gari SR Kloos H Alemu K. Barriers of persistent long-lasting insecticidal nets utilization in villages around Lake Tana, Northwest Ethiopia: a qualitative study. BMC Public Health. (2019) 19:1–11. 10.1186/s12889-019-7692-2 31619208 PMC6796332

[10] Wakefield MA Loken B Hornik RC. Use of mass media campaigns to change health behaviour. Lancet. (2010) 376:1261–71. 10.1016/S0140-6736(10)60809-4 20933263 PMC4248563

[11] Dako-Gyeke P Amazigo U V Halpaap B Manderson L. Social innovation for health: engaging communities to address infectious diseases. Infect Dis Poverty. (2020) 9:1–4. 10.1186/s40249-020-00721-3 32682449 PMC7368681

[12] Nyonator FK Awoonor-Williams JK Phillips JF Jones TC Miller RA. The Ghana community-based health planning and services initiative for scaling up service delivery innovation. Health Policy Plan. (2005) 20:25–34. 10.1093/heapol/czi003 15689427

[13] Binka FN Nazzar A Phillips JF. The Navrongo community health and family planning project. Stud Fam Plann. (1995) 121–39. 10.2307/2137832 7570763

[14] Awoonor-Williams JK Bawah AA Nyonator FK Asuru R Oduro A Ofosu A. The Ghana essential health interventions program: a plausibility trial of the impact of health systems strengthening on maternal & child survival. BMC Health Serv Res. (2013) 13:1–12. 10.1186/1472-6963-13-S2-S3 23819518 PMC3668206

[15] Glozah F Asampong E Tabong PT-N Nwameme A Hornuvo R Chandi M. Creating interventions to transition long-lasting insecticide net distribution in Ghana. BMJ Open. (2022) 12:e063121. 10.1136/bmjopen-2022-063121 35649610 PMC9161095

[16] Braun V Clarke V. Using thematic analysis in psychology. Qual Res Psychol. (2006) 3:77–101. 10.1191/1478088706qp063oa

[17] Koenker H Keating J Alilio M Acosta A Lynch M Nafo-Traore F. Strategic roles for behaviour change communication in a changing malaria landscape. Malar J. (2014) 13:1–4. 10.1186/1475-2875-13-1 24383426 PMC3882285

[18] Briscoe C Aboud F. Behaviour change communication targeting four health behaviours in developing countries: A review of change techniques. Soc Sci Med. (2012) 75:612–21. 10.1016/j.socscimed.2012.03.016 22541798

[19] Jagosh J Macaulay AC Pluye P Salsberg JON Bush PL Henderson JIM. Uncovering the benefits of participatory research: implications of a realist review for health research and practice. Milbank Q. (2012) 90:311–46. 10.1111/j.1468-0009.2012.00665.x 22709390 PMC3460206

[20] Ghosh SK Patil RR Tiwari S Dash AP. A community-based health education programme for bio-environmental control of malaria through folk theatre (Kalajatha) in rural India. Malar J. (2006) 5:1–7. 10.1186/1475-2875-5-123 17173672 PMC1716174

[21] Leask CF Sandlund M Skelton DA Chastin SFM. Co-creating a tailored public health intervention to reduce older adults' sedentary behaviour. Health Educ J. (2017) 76:595–608. 10.1177/001789691770778529546181

[22] Scott J Kanyangarara M Nhama A Macete E Moss WJ Saute F. Factors associated with use of insecticide-treated net for malaria prevention in Manica District, Mozambique: a community-based cross-sectional survey. Malar J. (2021) 20:1–9. 10.1186/s12936-021-03738-7 33906642 PMC8077836

[23] Aberese-Ako M Magnussen P Ampofo GD Tagbor H. Health system, socio-cultural, economic, environmental and individual factors influencing bed net use in the prevention of malaria in pregnancy in two Ghanaian regions. Malar J. (2019) 18:1–13. 10.1186/s12936-019-2994-5 31718677 PMC6852762

[24] Baltzell K Harvard K Hanley M Gosling R Chen I. What is community engagement and how can it drive malaria elimination? Case studies and stakeholder interviews Malar J. (2019) 18:1–11. 10.1186/s12936-019-2878-8 31315631 PMC6637529

[25] Perry HB Zulliger R Rogers MM. Community Health Workers in Low-, Middle-, and High-Income Countries: An Overview of Their History, Recent Evolution, and Current Effectiveness. (2014) 35:399–421. 10.1146/annurev-publhealth-032013-182354 24387091

[26] Tseng YH Griffiths F De Kadt J Nxumalo N Rwafa T Malatji H. Integrating community health workers into the formal health system to improve performance: a qualitative study on the role of on-site supervision in the South African programme. BMJ Open. (2019) 9:e022186. 10.1136/bmjopen-2018-022186 30819698 PMC6398712

[27] Lusambili AM Nyanja N Chabeda SV Temmerman M Nyaga L Obure J. Community health volunteers challenges and preferred income generating activities for sustainability: a qualitative case study of rural Kilifi, Kenya. BMC Health Serv Res. (2021) 21:642. 10.1186/s12913-021-06693-w 34217281 PMC8254366

